# Inventory of medium- to large-sized wild mammals from District Nagar, Gilgit-Baltistan, Pakistan through camera-trap surveys

**DOI:** 10.3897/BDJ.13.e151009

**Published:** 2025-05-13

**Authors:** Shahab Ud Din, Joong Yeol Park, Mihyeon Kim, Sungwon Hong

**Affiliations:** 1 Department of Animal Biotechnology Science, Kyungpook National University, Sangju, Republic of Korea Department of Animal Biotechnology Science, Kyungpook National University Sangju Republic of Korea; 2 Department of Horse/Companion, and Wild Animal Science, Kyungpook National University, Sangju, Republic of Korea Department of Horse/Companion, and Wild Animal Science, Kyungpook National University Sangju Republic of Korea

**Keywords:** Camera trapping, mammals, Gilgit-Baltistan, wildlife, conservation

## Abstract

**Background:**

The study area is located within the Central Karakoram National Park, nestled in the towering Karakoram mountain range. Situated at high altitude in the District of Nagar, Gilgit-Baltistan, Pakistan, this region is characterised by its rugged terrain, glaciers and snow-covered peaks. The area encompasses diverse habitats, including alpine meadows, subalpine forests and glaciated zones, which provide potential habitat for medium- to large-sized mammals. Despite its ecological significance, wildlife in this area remains understudied. Therefore, the current study aimed to document the wild mammal species of the high-altitude Hoper and Hisper Valleys.

**New information:**

Camera-trapping surveys were conducted from April to November 2024 to record wild medium- to large-sized mammals in the study area. Thirty trail cameras were deployed across the study area, which resulted in 85 occurrence records for seven mammal species, including three carnivores, two lagomorphs, one rodent and one ruminant. All species were native, non-endemic to the region. According to the IUCN Conservation Status, *Pantherauncia* (Snow Leopard), *Mustelaaltaica* (Mountain Weasel) and *Caprasibirica* (Himalayan Ibex) belong to the endangered categories. Out of the seven mammals recorded in this study, five are newly documented, while the Snow Leopard and Himalayan Ibex have been previously reported from the area. The data collected from this survey provide valuable occurrence information on the mammal species present in the study area, which can serve as a baseline for monitoring and conservation efforts in the future.

## Introduction

Medium- to large-sized mammals are often regarded as flagship and charismatic species, playing a vital role in the conservation of their ecosystems and habitats (Sasidhran et al. 2016). These animals are especially susceptible to human-related disturbances because of their expansive home ranges, limited population sizes, human-driven threats and reliance on pristine habitats for survival (Meyer et al. 2015). A deeper understanding of their distribution, behaviour and ecological requirements will enhance the baseline knowledge necessary for developing effective conservation strategies that safeguard both the species and the ecosystem they inhabit (Li et al. 2018). Given the increasing pressures on natural habitats, acquiring such knowledge has become more important.

Camera trapping is a non-invasive and effective method for studying wildlife, allowing researchers to document the presence, distribution and behaviour of mammals (Park et al. 2018, Delisle et al. 2021, Lim et al. 2023). This technique is particularly valuable in remote and rugged areas, such as Gilgit-Baltistan in Pakistan, where direct observations are challenging due to difficult terrain, elusive behaviour and likely lower densities of target species.

The study area, part of the Central Karakoram National Park (CKNP), is home to diverse ecosystems, including high-altitude valleys, glaciers and snow-covered mountains (Central Karakoram National Park 2025). Despite its ecological significance, there is still limited information on the wildlife populations in this region, especially medium- to large-sized mammals. This study aims to document and monitor wild mammal species through camera-trap surveys. The absence of comprehensive wildlife studies in this region, especially regarding the diversity and distribution of mammal species, results in a notable knowledge gap.

While previous studies, such as Nawaz et al. (2021) on snow leopard sampling design, Ali et al. (2015) on the Himalayan Ibex and Abbas (2015) in the "Winter Report on Seasonal Wildlife in CKNP," have focused on specific species, the presence of other mammal species remains largely unexplored. This underscores the need for further research to fully understand the area's biodiversity and assess the conservation status of its wildlife.

The current study used camera-trap surveys to provide critical baseline data on medium- to large-sized wild mammals, supporting conservation efforts for future wildlife management strategies.

## General description

### Purpose

The study aimed to document medium-large-sized mammals in the Hoper and Hisper valleys using camera-trapping data. The study provides baseline data for the unique wild mammal species of the study area.

## Project description

### Title

Study of Population and Distribution of Mammals in the high altitude valleys of Karakoram, Gilgit-Baltistan, Pakistan

### Personnel

Shahab Ud Din, Sungwon Hong

### Study area description

**Figure 1.**
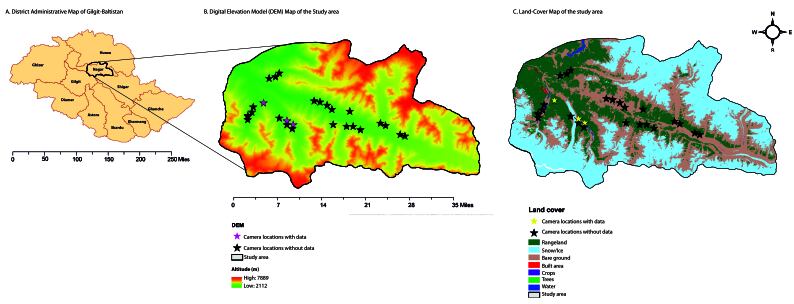
Map of Gilgit-Baltistan showing its districts, along with the Digital Elevation Model (DEM) and Land-Cover map of the study area. Camera locations are marked with stars: pink stars on the DEM map and yellow stars on the land-cover map represent the camera locations from which data were collected. Two of these cameras, placed less than 500 m apart, appear as a single location on the map, resulting in three locations being shown instead of four. Locations without data are marked with black stars.

**Figure 2.**
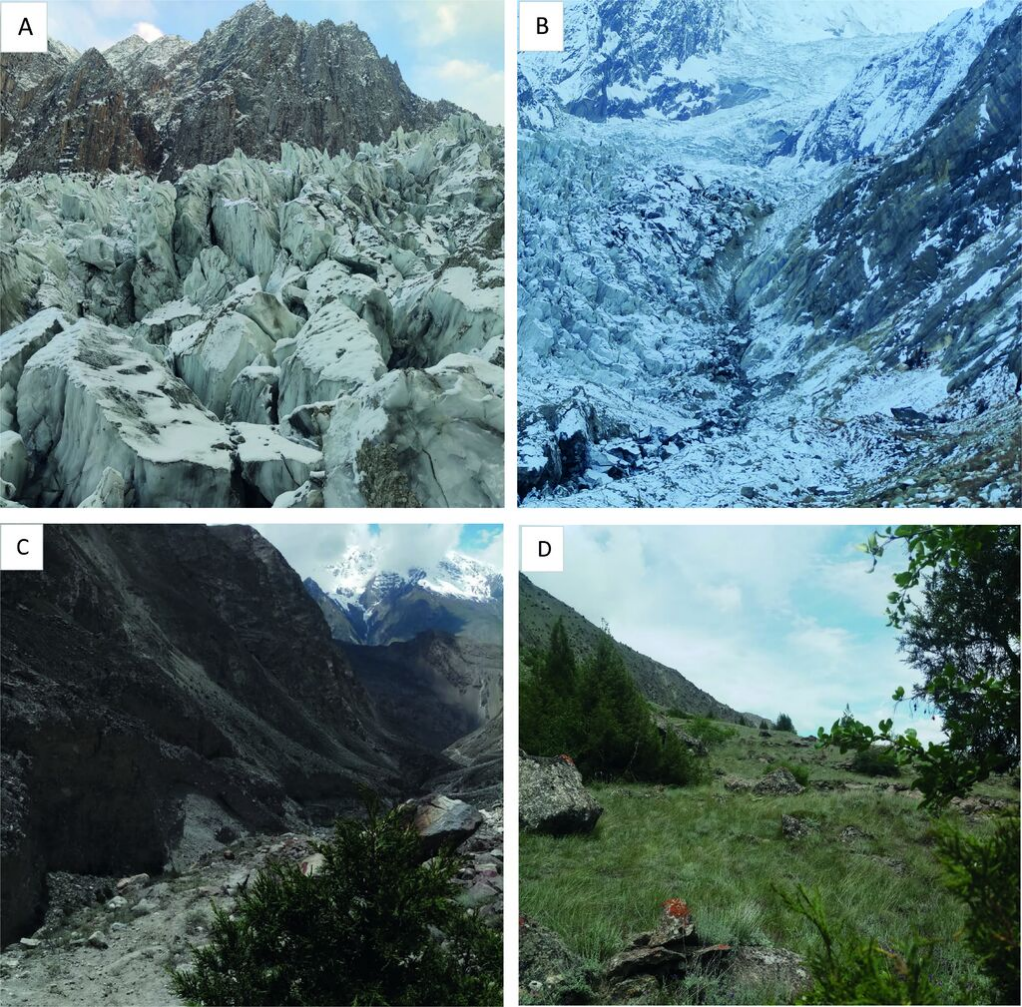
Images of the study area: **A, B** glacier; **C** dry mountainous area; **D** sub-alpine pasture. Pictures were taken during field surveys.

The study was conducted in the Hoper and Hisper Valleys, located in the Nagar District of Gilgit-Baltistan, Pakistan (Fig. 1). These valleys are part of the Central Karakoram National Park (CKNP) within the Karakorum Mountain Range. Encompassing an area of 1,735.84 km², the study area is characterised by its diverse topography, which includes watersheds, glaciers, towering snow-covered mountains and steep slopes (Nawaz et al. 2021). Fig. 2 presents images showcasing the landscape of the study area. Hoper Glacier (30 km) and Hisper Glacier (49 km) are the prominent glaciers in the study area. The study area spans temperate to sub-alpine and alpine zones with elevations ranging from 2,112 to 7,889 m (see Fig. 1). The climate is influenced by altitude, with winters being cold and dry, experiencing temperatures between −23°C and 10.8°C. In contrast, the summer months (June to August) are relatively mild, with temperatures reaching as high as 34°C. Precipitation in the study area is predominantly snowfall, accounting for about 90% of the total, with annual amounts ranging from 200 to 900 mm (Nawaz et al. 2021).

Both valleys contain lush green pastures that support a wide range of wild and domesticated animals, including goats, sheep, yaks and numerous wild ungulates. The diverse habitats and varying altitudes in these valleys may offer suitable conditions that support several wildlife species.

### Design description

The study area was divided into 2 km x 2 km grids (Tobler et al. 2008). Then, the areas lacking wildlife activity, including settlements, agricultural lands, glaciers and altitudes above 4500 m, were excluded from the sampling design. Camera sites were selected, based on local knowledge, with input from the community to identify areas of high wildlife activity. The presence-absence data were collected for medium-large mammal species.

### Funding

This research was supported by the Sejong Science Fellowship of the National Research Foundation (NRF) [grant number NRF-2021R1C1C2004162].

## Sampling methods

### Study extent

The current study recorded the occurrence of medium-large mammals over eight months. The camera trap locations spanned an altitudinal gradient ranging from 2,704 to 3,989 m above sea level.

### Sampling description

Thirty trail cameras (T-9 Pro Black) were installed across the study area to record medium- to large-sized mammals. The cameras were placed in areas of known wildlife activity, such as animal trails, water sources and feeding zones, ensuring a representative sample across different elevations. The survey was conducted in two sessions: the first session took place in April 2024, with data retrieval in June 2024. After data retrieval in June, the cameras were maintained by replacing the batteries and SD cards, then left in place until November 2024. In cases where no wildlife was detected, camera locations were adjusted and repositioned within a 500-m radius of the original site to improve the likelihood of capturing wildlife activity. Once deployed, camera traps operated continuously for 24 hours per day, with each operational day counted as one trap day. Only independent detections (i.e. images of the same species at the same camera location separated by at least 30 minutes) were used for analysis to avoid pseudo-replication. The sampling effort was calculated by summing the number of days each functioning camera trap remained operational, resulting in 396 camera-trap days.

### Quality control

To ensure accurate species identification, the authors thoroughly reviewed the photos and cross-checked the nomenclature, consulting the IUCN Red List (IUCN 2025) and also by considering each species' conservation status.

### Step description

**Figure 3.**
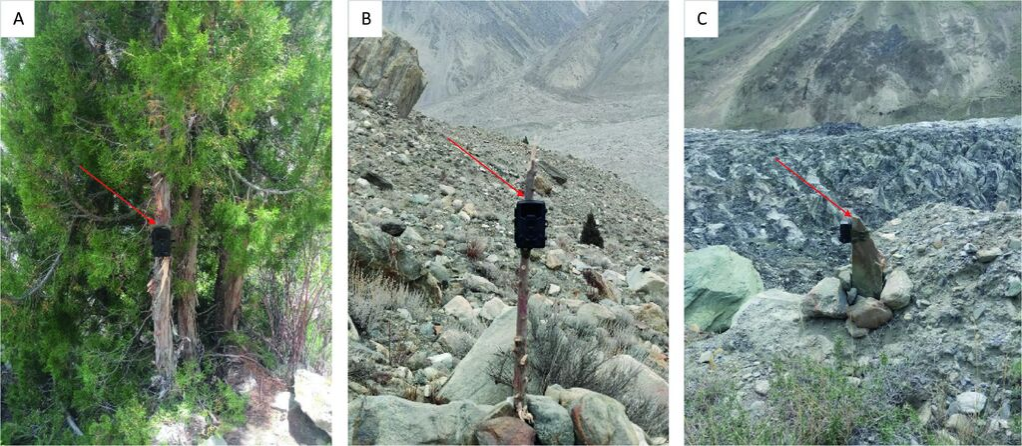
Camera traps installed in the study area: **A** attached to a tree trunk; **B** attached to a wooden stick; **C** tied on a stone.

At each site, the camera was installed on a tree trunk, rock or wooden stick (Fig. 3), depending on the available structures (Srbek-Araujo and Chiarello 2013, Lamelas-Lopez et al. 2020). The direction and angle of the camera were adjusted according to the terrain and expected wildlife movement patterns, ensuring a wide field of view to capture wildlife activity while minimising the chances of false triggers. After installation, any barriers in front of the camera, such as vegetation or tree branches, were removed to prevent false triggers and ensure reliable image capture (Oberosler et al. 2017).

The trail cameras were set as follows: the cameras remained active for 24 hours, with three photos per trigger and a quiet period of 30 seconds between triggers (Lamelas-Lopez et al. 2020). The sensitivity was set to medium to ensure reliable wildlife detection and minimise false triggers caused by wind, moving vegetation and temperature fluctuations, particularly in high-altitude areas. The camera name was customised, based on the site name (e.g. HP01, HP02) to facilitate easy identification and the date and time settings were correctly configured to ensure accurate time-stamping of all images (Oberosler et al. 2017).

The images were manually classified and renamed after retrieving data from the camera traps. Subsequently, using XnView software, the date and time were extracted from the images. The data were organised as a sample event dataset, encompassing both the event and occurrence data and published as a Darwin Core Archive (DwC-A) (Zuleger et al. 2023).

## Geographic coverage

### Description

Hoper Valley, situated 3,000 m above sea level and Hisper, located 3,500 m above sea level, were covered.

### Coordinates

36.16084 and 36.20245 Latitude; 74.78908 and 74.85652 Longitude.

## Taxonomic coverage

### Description

In this study, we covered the following taxonomic groups: Class: Mammalia; Orders: Carnivora, Lagomorpha, Rodentia and Artiodactyla; Families: Felidae, Canidae, Mustelidae, Bovidae, Leporidae, Ochotonidae and Muridae (Table 1).

## Temporal coverage

**Data range:** 2024-4-15 – 2024-11-15.

### Notes

The data were collected during the specified period.

## Collection data

### Collection name

Inventory of wild mammals from District Nagar, Gilgit-Baltistan, Pakistan

### Collection identifier

https://doi.org/10.15468/a8gt5r

## Usage licence

### Usage licence

Creative Commons Public Domain Waiver (CC-Zero)

## Data resources

### Data package title

Inventory of wild mammals from District Nagar, Gilgit-Baltistan, Pakistan

### Resource link

https://doi.org/10.15468/a8gt5r

### Alternative identifiers

https://cloud.gbif.org/asia/resource?r=wildlife_nagar

### Number of data sets

2

### Data set 1.

#### Data set name

Event table

#### Data format

Darwin Core Archive

#### Character set

UTF-8

#### Download URL

https://www.gbif.org/dataset/99aaf87c-d4a5-4239-a42a-63df6eb7dbe9

#### Data format version

Version 1.3

#### Description

The dataset is publicly available on the Global Biodiversity Information Facility (GBIF) platform (Din and Hong 2025). It includes species-level records, structured as a sample event dataset. The dataset is organised into two primary tables: the event table and the occurrence table. The data in this sampling are presented as an event table, which contains 25 records. The data are shared in the Darwin Core Archive (DwCA) format, a standardised structure that facilitates the sharing of biodiversity data across platforms. The dataset is archived using the Integrated Publishing Toolkit (IPT), which serves as the data repository, ensuring its long-term availability. The complete dataset, along with the associated resource metadata, can be downloaded from Din and Hong (2025).

**Data set 1.** 

Column label	Column description
eventID	Identifier of the events, unique for the dataset.
samplingProtocol	The sampling method used to obtain the records.
sampleSizeValue	The number of days that the cameras remain active in each sampling.
sampleSizeUnit	The unit of the sample size value.
samplingEffort	The amount of effort or time spent collecting the sample.
eventDate	Date or date range the record was collected.
eventTime	The specific time of day when the event or sampling occurred.
habitat	The habitat type in which the event occurred.
parentEventID	The specific event (survey) during which the data were collected.
locationID	Identifier of the location.
country	Country of the sampling site.
countryCode	ISO code of the country of the sampling site.
stateProvince	Name of the region of the sampling site.
decimalLatitude	The geographic latitude, in decimal degrees.
decimalLongitude	The geographic longitude, in decimal degrees.
geodeticDatum	The ellipsoid, geodetic datum or spatial reference system (SRS) upon which the geographic coordinates given in decimalLatitude and decimalLongitude are based.
coordinateUncertaintyInMetres	Uncertainty of the coordinates, in metres.

### Data set 2.

#### Data set name

Occurrence Table

#### Data format

Darwin Core Archive

#### Character set

UTF-8

#### Download URL

https://www.gbif.org/dataset/99aaf87c-d4a5-4239-a42a-63df6eb7dbe9

#### Data format version

Version 1.3

#### Description

The dataset is publicly available on the Global Biodiversity Information Facility (GBIF) platform (Din and Hong 2025). It includes species-level records, structured as a sample event dataset. The dataset is organised into two primary tables: the event table and the occurrence table. The data are shared in the Darwin Core Archive (DwCA) format, which is a widely accepted, standardised format for biodiversity data designed to facilitate the sharing of information across platforms and research initiatives. Within this dataset, the occurrence table contains 85 individual records that document specific organism occurrences. The dataset is archived using the Integrated Publishing Toolkit (IPT), which serves as the repository for the data, ensuring its availability for future use. The complete dataset, along with the associated resource metadata, can be downloaded from Din and Hong (2025).

**Data set 2.** 

Column label	Column description
occurrenceID	Identifier of the record, coded as a global unique identifier.
basisOfRecord	The nature of the data record.
organismQuantity	A number or enumeration value for the quantity of organisms.
organismQuantityType	The type of quantification system used for the quantity of organisms.
occurrenceStatus	Information about the presence/absence of a taxon at a camera location.
eventID	Identifier of the events, unique for the dataset.
eventDate	Date or date range the record was collected.
eventTime	The specific time of day when the event or sampling occurred.
scientificName	Complete scientific name including author and year.
kingdom	Kingdom name.
class	Class name.
order	Order name.
family	Family name.
genus	Genus name.
specificEpithet	Specific epithet.
taxonRank	Lowest taxonomic rank of the record.
sex	Sex of the organism.

## Additional information

Out of the 30 cameras deployed, 18 were excluded from the analysis due to various issues, such as lost cameras (possibly due to theft or animal interference), missing SD cards or malfunctioning equipment. Of the remaining 12 cameras that were operational, eight were triggered randomly and had full SD cards within two days, leaving only four cameras that successfully recorded wildlife data. While the cameras captured images of reptiles, birds and livestock (including goats, sheep and yaks), only the data related to wild mammals were included in this study.

**Figure 4.**
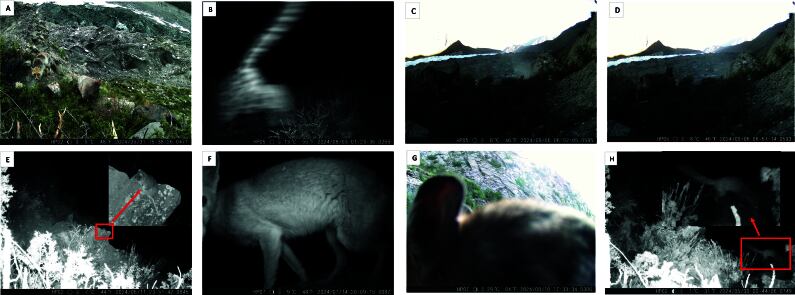
Example images of species captured by the camera trap: **A** Red fox; **B** Snow leopard; **C** Himalayan ibex (male); **D** Himalayan ibex (female); **E** Turkestan rat; **F** Cape hare; **G** Ladakh pika; **H** Mountain weasel. All images are date- and time-stamped.

A total of 85 occurrence records of wild mammals were obtained; all belonged to the class Mammalia, spanning four orders, seven families and seven species (Table 1). Fig. 4 displays sample images of each species captured by camera traps. Amongst the seven species, the Red fox (*Vulpesvulpes*), Cape hare (*Lepuscapensis*) and Ladakh pika (*Ochotona Ladakhiana)* are categorised as Least Concern (LC) by the IUCN, indicating their conservation status is stable both globally and in Pakistan. However, species such as the snow leopard (*Pantherauncia)*, which is classified as vulnerable (VU) and the Himalayan ibex (*Caprasibirica*) and mountain weasel (*Mustelaaltaica)*, which are listed as near-threatened (NT), face higher risks of extinction and, therefore, need greater conservation attention. All species are native, non-endemic to Pakistan. In this study, the Red Fox accounted for the highest number of occurrences (42), while others like the Snow leopard and the Turkestan rat were recorded only three times each, indicating they may occur at lower densities or be less detectable in this landscape (Table 2). These data highlight the diversity of mammal species in Hoper and Hisper Valleys of Gilgit-Baltistan, Pakistan and underscore the need for ongoing conservation efforts to protect the more vulnerable species.

However, this study yielded a relatively low number of wildlife records, largely due to a combination of technical and operational challenges. As mentioned earlier, key contributing factors included the loss of cameras, missing SD cards, incorrect settings configurations and camera malfunctions. Similar difficulties have been reported in other regions. For instance, Foster et al. (2021) encountered comparable issues in New Zealand; however, their use of a greater number of camera traps, longer deployment periods and different camera models led to higher detection rates than ours. Sathyakumar et al. (2011) also identified camera failures due to extreme temperatures, rugged terrain and environmental interference such as wind-induced triggers, as well as the loss of equipment, as major limitations in their study.

Comparable challenges have been documented across the Himalayan Region. Lama et al. (2024) reported data loss resulting from snowfall, theft, human error and livestock disturbances. Banjade et al. (2024) highlighted that a short survey period contributed to limited detections, while consistently low detection probabilities (< 0.2) have been observed across Nepal, India, Bhutan and Bangladesh (Ghimirey et al. 2024).

Beyond these methodological issues, the low detection rates in our study may also reflect genuinely low densities of medium- to large-sized mammals. Jiménez et al. (2010), for example, reported only 313 images of eight mammal species and attributed the low capture rate to sparse populations. Although their study was conducted in a different region, the findings underscore a broader point: in remote, high-altitude environments, low detection rates may be more indicative of naturally low animal densities than merely technical shortcomings. In our study, detections of livestock were higher compared to those of wild mammals, even during periods when the cameras were confirmed to be functioning properly. This contrast may suggest that wildlife densities are significantly lower than those of domestic animals, providing indirect insight into relative abundance patterns.

Collectively, these patterns suggest that low detection rates, driven by both technical challenges and genuinely low wildlife densities, are a common issue in mountainous and ecologically extreme landscapes. Therefore, future surveys should incorporate longer deployment periods, improved camera settings and more robust sampling designs to better address these limitations and improve detection outcomes.

## Figures and Tables

**Table 1.** Taxonomic groups covered in the current study.

**Rank**	**Scientific Name**	**Common Name**
class	Mammalia	Mammals
order	Carnivora	Carnivores
order	Lagomorpha	Hares, Pikas
order	Rodentia	Rodents
order	Artiodactyla	Ruminants
family	Felidae	Cats
family	Canidae	Canids
family	Mustelidae	Mustelids
family	Bovidae	Bovid
family	Leporidae	Hares
family	Ochotonidae	Pikas
family	Muridae	Murids

**Table 2.** Mammals and their order, species, common names, IUCN status, status within Pakistan, colonisation status and occurrence records. NT - Near Threatened; LC - Least Concern; VU - Vulnerable; nat - native non-endemic.

**Order**	**Family**	**Species**	**Common name**	**IUCN**	**Conservation status (Pakistan)**	**Colonisation status**	**No. of occurrences**
Carnivora	Felidae	*Pantherauncia* (Schreber, 1775)	Snow leopard	VU	VU	nat	3
Carnivora	Canidae	*Vulpesvulpes* (Linnaeus, 1758)	Red fox	LC	LC	nat	42
Carnivora	Mustelidae	*Mustelaaltaica* (Pallas, 1811)	Mountain weasel	NT	LC	nat	10
Artiodactyla	Bovidae	*Caprasibirica* (Pallas, 1776)	Himalayan ibex	NT	LC	nat	9
Lagomorpha	Leporidae	*Lepuscapensis* (Linnaeus, 1758)	Cape hare	LC	LC	nat	6
Lagomorpha	Ochotonidae	*Ochotonaladacensis* (Günther, 1875)	Ladakh pika	LC	LC	nat	12
Rodentia	Muridae	*Rattusturkestanicus* (Satunin, 1903)	Turkestan rat	LC	LC	nat	3
